# Genome Mining-Based Discovery of Blenny Fish-Derived Peptides Targeting the Mouse κ-Opioid Receptor

**DOI:** 10.3389/fphar.2021.773029

**Published:** 2021-10-22

**Authors:** Edin Muratspahić, Bernhard Retzl, Leopold Duerrauer, Michael Freissmuth, Christian F. W. Becker, Christian W. Gruber

**Affiliations:** ^1^ Center for Physiology and Pharmacology, Institute of Pharmacology, Medical University of Vienna, Vienna, Austria; ^2^ Institute of Biological Chemistry, Faculty of Chemistry, University of Vienna, Vienna, Austria; ^3^ Gaston H. Glock Research Laboratories for Exploratory Drug Development, Center for Physiology and Pharmacology, Medical University of Vienna, Vienna, Austria

**Keywords:** genome mining, GPCR, drug discovery, opioid, peptide

## Abstract

Over the past years, peptides have attracted increasing interest for G protein-coupled receptor (GPCR) drug discovery and development. Peptides occupy a unique chemical space that is not easily accessible for small molecules and antibodies and provide advantages over these ligand classes such as lower toxicity and higher selectivity. The κ-opioid receptor (KOR) is a prototypic GPCR and an appealing therapeutic target for the development of safer and more effective analgesics. Recently, peptides have emerged as analgesic drug candidates with improved side effect profiles. We have previously identified plant-derived peptides, which activate KOR. Based on this precedent, here we relied on publicly available databases to discover novel KOR peptide ligands by genome mining. Using human preprodynorphin as a query, we identified blenny fish-derived peptides, referred to as blenniorphins, capable of binding to and activating KOR with nanomolar affinity and potency, respectively. Additionally, the blenniorphins altered β-arrestin-2 recruitment at the KOR. Our study demonstrates the utility of genome mining to identify peptide GPCR ligands with intriguing pharmacological properties and unveils the potential of blenny fishes as a source for novel KOR ligands.

## Introduction

G protein-coupled receptors (GPCRs) are the largest, most versatile and most ubiquitous family of cell surface receptors involved in regulating a myriad of physiological processes (Lagerström and Schiöth, 2008; Rajagopal et al., 2010). They are amongst the most commonly targeted receptor classes: more than 475 approved drugs mediate their pharmacological effects by targeting these receptors (Hauser et al., 2017). Opioid receptors are prototypic class A GPCRs comprising four subtypes referred to as the κ-opioid receptor (KOR), µ-opioid receptor (MOR), δ-opioid receptor (DOR), and nociceptin receptor (NOP), which represents a related nonclassical opioid receptor (Che et al., 2021). They constitute primary targets for opioids including morphine and fentanyl, which continue to be the cornerstone of today’s pain therapy (Darcq and Kieffer, 2018). While being effective painkillers, their ability to target preferentially MOR, frequently results in the development of deleterious side effects including respiratory depression, nausea and constipation, as well as tolerance and addiction (Volkow and Collins, 2017). In fact, the use of opioids as analgesics for medical purposes and as illegal substances for drug misuse has recently escalated into a global “opioid crisis” thus affecting public health as well as social and economic welfare (Volkow and Collins, 2017; Darcq and Kieffer, 2018).

In that regard, KOR has received attention in the development of safer analgesics (Che et al., 2021). Its agonist-induced activation is devoid of adverse effects commonly observed for the modulation of MOR, and it is this characteristic that has sparked interest in targeting KOR to develop alternative opioid analgesics (Che et al., 2021). However, the activation of KOR results in the development of sedation, dysphoria and hallucinations, which limits the therapeutic potential of selective KOR agonists (Darcq and Kieffer, 2018). Thus, over the past years strategies have been developed to design ligands with reduced KOR-specific side effects (Machelska and Celik, 2018). One strategy is to search for biased ligands. These agonists preferentially activate either the G protein-dependent or the β-arrestin-dependent signaling pathways. It is based on the tenet that side effects of KOR arise predominantly from signaling pathways initiated by the recruitment of β-arrestin (Che et al., 2021). However, despite the development of numerous biased ligands with promising therapeutic potential, biased signaling remains controversial in the field of opioid receptors (White et al., 2015; Brust et al., 2016a; Schattauer et al., 2017). The second strategy is to restrict the action of agonists to the peripheral nervous system by generating ligands with limited capability to penetrate the central nervous system. This has proven effective in developing analgesics without centrally mediated side effects (Snyder et al., 2018). In particular, peptide ligands targeting KOR expressed in the peripheral nervous system have demonstrated therapeutic potential (Hughes et al., 2013; Snyder et al., 2018; Beck et al., 2019). This can be gauged from the recent approval of the tetrapeptide difelikefalin for the treatment of postsurgical abdominal pain. Difelikefalin acts as a selective, peripherally restricted KOR agonist (Davenport et al., 2020). It is noteworthy that the two strategies are not mutually exclusive. The discovery of novel peptide ligands of KOR is not only of therapeutic interest, but they may also allow for linking the effect of KOR activation at distinct sites *in vivo* to the stimulation of different signaling cascades. Nature-derived peptides can be exploited for discovery of GPCR ligands (Muratspahić et al., 2019). This strategy was successfully applied to identify KOR ligands in plants: inspired by the traditional use of sunflower seed extracts as analgesic and anti-inflammatory remedy, we recently discovered and developed a potent, G protein biased and selective KOR peptide ligand as potential abdominal pain therapeutic.

Ethnopharmacology has inspired current drug discovery efforts (Atanasov et al., 2021). Alternatively, genome mining has emerged as powerful strategy for the discovery of novel nature-derived peptides targeting GPCRs. For example, recent analysis of publicly available genome and transcriptome datasets of insect species facilitated the identification of a plethora of oxytocin/vasopressin-like peptides (Gruber and Muttenthaler, 2012; Liutkeviciute et al., 2016). Applying this strategy enabled the development of an arthropod-derived G protein-biased peptide ligand of the V2R termed I8-arachnotocin (Duerrauer et al., 2019) as well as the design of a potent, selective and competitive oxytocin/vasopressin-like V1aR antagonist, referred to as inotocin, derived from the black garden ant *Lasius niger* (Di Giglio et al., 2017).

In the present study, we employed a genome mining approach for the discovery of fish-derived peptides based on their sequency homology to the endogenous KOR peptide dynorphin (dyn) A 1–17 (Chavkin and Goldstein, 1981; Chavkin et al., 1982). The identified blenny fish-derived peptides, referred to as blenniorphins, were prepared by solid-phase peptide synthesis (SPPS). The resulting peptides were applied to radioligand binding and functional second messenger and β-arrestin-2 recruitment assays to explore their pharmacological properties at the KOR. Blenniorphins demonstrated the ability to bind to and activate KOR with nanomolar affinity and potency. Therefore, our study reinforces the power of genome mining and blenny fishes as a source to identify potent peptide KOR ligands.

## Materials and Methods

### Materials

Dyn A 1–13 amide trifluoroacetate salt was purchased from Bachem (Switzerland). Naloxone and forskolin were obtained from Sigma-Aldrich (Austria) and [^3^H]-diprenorphine was from Perkin Elmer (Austria). The cAMP Gi kit was from CisBio (France) and jetPRIME transfection reagent from Polyplus (Austria).

### Transcriptome Mining and Sequence Analysis

For initial similarity analysis we accessed the National Center for Biotechnology Information (NCBI) BLAST online interface to search via SWISSPROT for dynorphin-like peptides using human preprodynorphin precursor as a query. For subsequent large scale data mining, fish transcriptome data sets available via the NCBI sequence set browser (Supplementary Table 1) were downloaded and conceptually translated in six frames using custom Python scripts. A BLAST database was created from all contigs containing the dynorphin characteristic YGGF motif. BLAST searches were conducted using human preprodynorphin precursor (UniProt ID: P01213) and all hits with an E (expect)-value smaller or equal to 10^–6^ were considered as significant. Only unique BLAST hits aligning to human dyn A 1–17 were retained for further sequence analysis. A novel sequence or an already identified sequence in a new species were considered as a unique hit. Sequence logos were created using WebLogo after clustering the hits into 79 taxonomic groups. All hits were clustered into a taxonomic profile according to Hughes and others (Hughes et al., 2018).

### Synthesis and Analytical Characterization of Peptides

Blenniorphins were produced using Fmoc-based SPPS as previously described (Duerrauer et al., 2019). Blenniorphin 1–17 was synthesized on a Fmoc-L-Ser(t-Bu) TentaGel R resin (Rapp Polymere, Germany) using 5-fold excess of protected amino acids, 5 equivalents of hexafluorophosphate benzotriazole tetramethyl uranium (HBTU) (Iris Biotech, Germany) and N,N-diisopropylethylamine (DIPEA) (Merck, Germany) in dimethylformamide (DMF) (Merck) and a coupling time of 30 min. Peptides were cleaved of the resin *via* treatment with trifluoracetic acid (TFA, 92.5%) (VWR, United States): H_2_O (2.5%): triisopropylsilane (TIPS, 2.5%) (Merck): ethandithiol (EDT, 2.5%) (Merck) for 3 h. Blenniorphin 1–8 and 1–13 were synthesized via Boc-based SPPS. The peptides were assembled on a preloaded Boc-Trp-OH PAM resin (Iris Biotech) using 10-fold excess of protected amino acids, 9.8 equivalents of HBTU and DIPEA in DMF, and a coupling time of 15 min. The peptides were cleaved of the resin via treatment with liquid hydrogen fluoride (10 ml) (Air Liquide, France) and p-cresol (0.5 ml per g of resin) (Merck) on ice for 1 h. All peptides were purified on a reverse-phase high-performance liquid chromatography (RP-HPLC) system using a linear gradient of 5–65% of solvent A consisting of H_2_O with 0.1% TFA to solvent B consisting of 90% acetonitrile (Merck), 10% H_2_O with 0.1% TFA in 60 min at a flow rate of 8 ml/min on a Phenomenex Jupiter C_18_ column (250 × 21.2 mm, 10 μm, 300 Å; Phenomenex, Germany). Peptide purity was analyzed by analytical RP-HPLC using a Kromasil C_18_ column (250 × 4.6 mm, 5 μm, 100 Å; dichrom GmbH, Germany) via a linear gradient 5–65% in 60 min at a flow rate of 1 ml/min and MALDI mass spectrometry. MALDI analysis of peptides was performed by MALDI-TOF/TOF 4,800 Analyzer (AB Sciex, United States) operated in reflector positive ion mode acquiring between 2,000 and 10,000 total shots per spectrum with a laser intensity set of 3,500. Peptide samples were prepared using α-cyano-hydroxyl-cinnamic acid as matrix (Sigma–Aldrich) in 50% H_2_O, 50% acetonitrile and 0.1% trifluoroacetic acid, at a concentration of 5 mg/ml. 0.5 μl of peptides were mixed with of 3 µl of matrix and spotted directly onto the MALDI target plate. Spectra were processed and analyzed using the Data Explorer SoftwareTM (AB Sciex).

### Cell Culture, Transfection and Cloning

Cell culture, transfection and cloning of mouse KOR have been performed as previously described (Muratspahić et al., 2021).

### Radioligand Displacement Binding Assays

HEK293 cell membranes stably expressing mouse KOR were prepared as previously described (Nasrollahi-Shirazi et al., 2020) and were used in radioligand displacement binding assays as described in Muratspahić *et al.* (Muratspahić et al., 2021) Briefly, the assay was carried out in duplicates in a final volume of 300 µl containing binding buffer (50 mM Tris-HCl pH 7.4, 10 mM MgCl_2_, and 0.1% bovine serum albumin), [^3^H]-diprenorphine (1 nM final concentration), peptides (logarithmically spaced concentrations covering the range of 0.1 nM–30 µM) and membranes (6.3–7.6 µg/assay). The reaction mixture was incubated for 1.5 h at 37°C. Nonspecific binding was determined in the presence of 10 µM naloxone. Bound ligand was separated from free ligand by rapid filtration using 0.1% polyethlyenimine-soaked GF/C glass fiber filter and a Skatron cell harvester.

### cAMP Assay

Cyclic AMP (cAMP) accumulation by HEK293 cells stably expressing mouse KOR was measured in triplicate with using the homogenous time-resolved fluorescence resonance energy transfer (HTRF) cAMP–Gi kit according to the manufacturer’s protocol with minor modifications. Briefly, cells (2,000/well) were seeded into white 384-well plates and incubated in a final volume of 10 µl containing logarithmically spaced concentrations of peptides covering the range of 0.03 nM–30 μM, in stimulation buffer supplemented with IBMX (0.5 mM final concentration) and forskolin (10 µM final). The reaction mixture was incubated at 37°C for 30 min. After cell lysis, Europium cryptate-labeled cAMP and cAMP d2-labeled antibody were added and, the reaction mixtures were incubated for 1 h at room temperature. Subsequently, cAMP levels were measured by HTRF on a Flexstation 3 (Molecular Devices, United States) using the ratio of 665/620 nm.

### β*-*Arrestin-2 Recruitment Assay

Bioluminescence resonance energy transfer (BRET)-based β-arrestin-2 recruitment assays were conducted on HEK293 cells transiently co-expressing human β-arrestin-2-Nluc and mouse KOR-EGFP in a 1:10 ratio. Sixteen to 24 h after transfection, cells were transferred into white, clear-bottom cell culture plates in phenol red-free Dulbecco’s Modified Eagle Medium (DMEM) supplemented with 10% fetal bovine serum at a density of 50,000 cells/well and incubated overnight at 37°C. The next day, cells were serum starved for 1 h at 37°C in phenol red-free DMEM. (HBSS). The assay was performed in duplicate: furimazine (50 µl of a 1:50 dilution) was added to the cells and incubated for 5 min at 37°C. After establishing the baseline for 5 min, ligands (diluted in Hank’s balanced salt solution) were automatically added, and the response was measured for 35 min. The plates were measured for both luminescence at 460 nm for Nluc and fluorescence at 510 nm for EGFP using the Flexstation 3.

### Pharmacological Data Analysis

Data analysis was performed using GraphPad Prism (GraphPad Software, United States). Concentration response curves were generated by fitting the data obtained in cAMP assays to the equation for a monophasic inhibition to obtain potency (EC_50_) and maximum efficacy (E_max_). Data were normalized to an effect size of 100%, which corresponds to the inhibition elicited by the highest concentration of the positive control dyn A 1–13 (10 µM) used in the assay. The ligand-induced BRET signal was calculated as: (emission EGFP_ligand_/emission Nluc_ligand_)—(emission EGFP_HBSS_/emission Nluc_HBSS_). Relative BRET quantification was determined by calculating the mean value of ligand-induced BRET for blenniorphins from 311 to 2,411 s (35 min). This value was divided by the mean value of BRET induced by dyn A 1–13 (10 µM), which was used as reference value to normalize for inter-assay variation. IC_50_-values were calculated by fitting the data obtained in radioligand displacement binding assays to a three-parameter logistic Hill equation and converted to inhibition constants (*K*_i_) using the Cheng and Prusoff approximation. *K*_d_ (0.87 ± 0.06 nM) and Bmax (7,166 ± 147 fmol/mg protein) values of [^3^H]-diprenorphine were previously determined (Muratspahić et al., 2021). Data were expressed in counts per min (cpm) of total binding of the radioligand, whereas 1 cpm ≙ 38.4 fmoles. Nonspecific binding was determined from the curve fit and experimentally.

## Results

### Genome Mining

**FIGURE 1:**
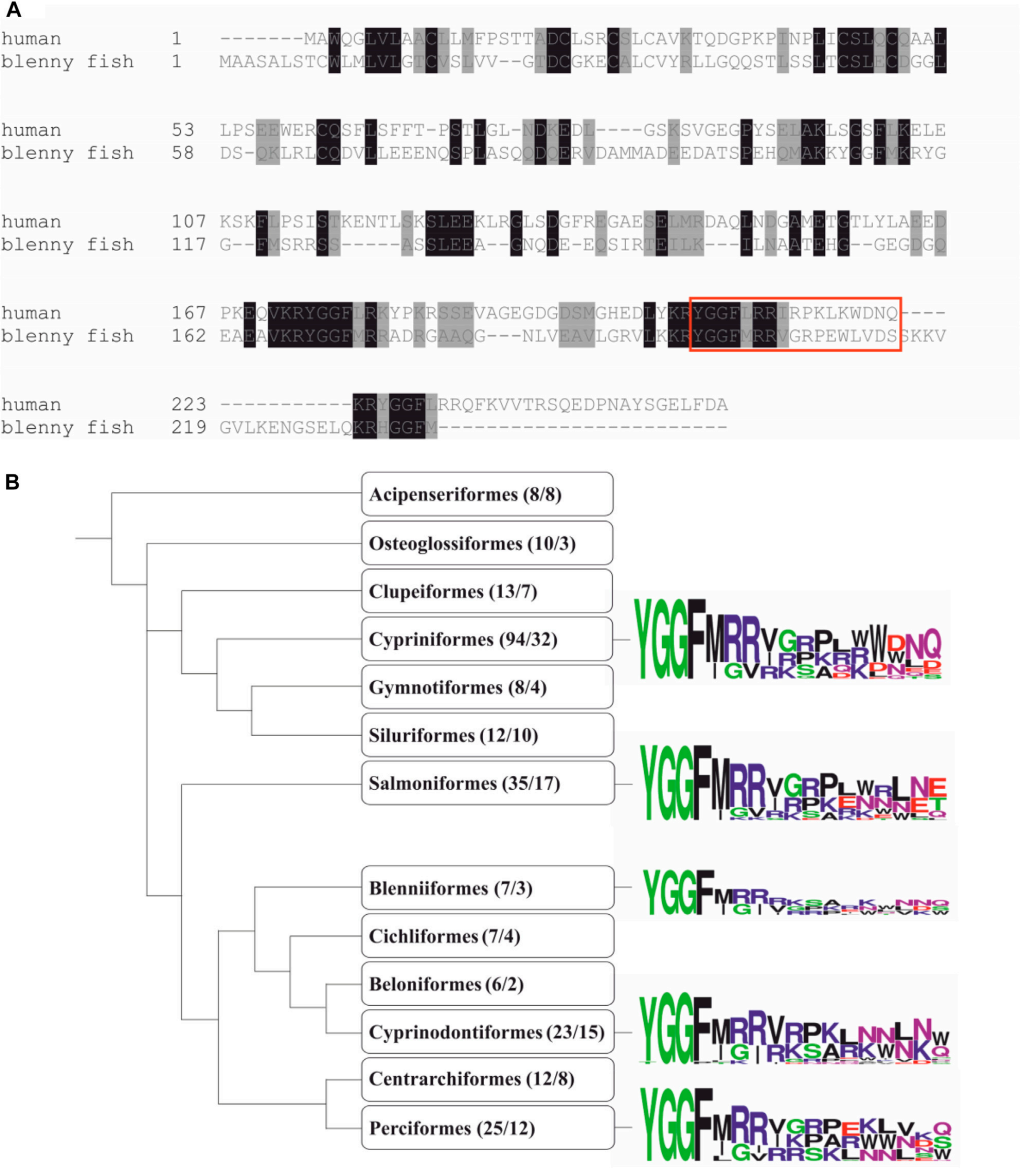
Genome mining. **(A)** Comparison of the human preprodynorphin precursor (Sequence ID: P01213 and the novel precursor identified in the blenny fish *M. atrodorsalis*. The identified hit (Sequence ID: P0DP56.1) contains the YGGF motif typical for human opioid peptides. FASTA sequences were aligned by Clustal Omega and are shown in box-shade format. Identical and similar amino acid residues are highlighted in black and grey, respectively. Mature human dyn A 1–17 and blenny fish dyn A 1–17-like peptide are indicated by the red box. **(B)** A schematic phylogenetic tree based on the taxonomic profile of fish as described by Hughes et al. (2018) is illustrated. Only taxa with more than five hits are shown. The numbers of unique hits per taxonomic group and the number of available transcriptomes in this group are displayed in brackets, separated by a forward slash, respectively. Sequence logos of hits were generated with WebLogo for the taxa Cypriniformes, Salmoniformes, Cyprinodontiformes, and Perciformes. They are shown as representative examples in comparison to Blenniiformes.

Mining of publicly available databases such as provided by the NCBI provides an opportunity for the discovery of novel gene-derived peptides. Non-mammalian opioid peptides active at mammalian opioid receptors were isolated, for instance, from frogs (Kreil, 1994), scorpions (Zhang et al., 2012), cone-snails (Brust et al., 2016b), fungi (Dekan et al., 2019), and nematodes (Cheong et al., 2015). In contrast, fish-derived opioid peptides targeting KOR have not yet been reported. Accordingly, we performed a BLASTP (Protein Basic Local Alignment Search Tool) search against fish proteins available in the UniProtKB/SWISSPROT database by using the human preprodynorphin precursor as a query. Intriguingly, this search resulted in the identification of a single hit with an E-value of 3 × e^-12^ (Figure 1A). The observed precursor hit was found in the transcriptome of the blenny fish *Meicanthus atrodorsalis* and it contained the YGGF motif typical for endogenous human opioid peptides (Naqvi et al., 1998). This finding prompted us to explore the distribution and sequence variation of the fish dynorphin system by extending our search to fish transcriptome data. We downloaded all available 379 bony fish transcriptome data sets listed on the NCBI sequence set browser (Supplementary Table 1) comprising transcriptome records of 200 different fish species. Subsequently, we conceptually translated these records in all six reading frames using custom Python scripts and selected all 201937 contigs containing the YGGF motif for BLAST analysis. The BLAST search using human preprodynorphin precursor as a query yielded 571 hits aligning to the human preprodynorphin precursor with an E-value of 10^–6^ or less (Supplementary Table 2). After removing non-unique BLAST hits as described in the Methods section 338 hits were retained. In 146 species (73%) we identified hits in the preprodynorphin BLAST analysis. Figure 1B summarizes a selection (i.e., number of hits >5 per group) of the taxonomic orders of bony fish that contain human dynorphin-like sequences. In addition, the mature dynorphin-like sequences were extracted for four orders with the greatest number of hits (i.e., Cypriniformes, Salmoniformes, Cyprinodontiformes, and Perciformes, in comparison to Blenniiformes). The taxonomic profile of fish was prepared as described by Hughes et al. (2018) and it contains 79 different taxa. For 41 taxa (52%) transcriptome datasets are available and in 39 of the 41 taxa hits were identified. Overall, our *in silico* analysis is indicative for a ubiquitous and taxa-wide distribution of dynorphin precursor sequences in fish, and large diversity of mature peptides (Figure 1B). The number of available transcriptomes per taxonomic group and the number of unique hits per group is provided in Supplementary Table 3.

### Chemical Synthesis of Blenniorphins

**FIGURE 2:**
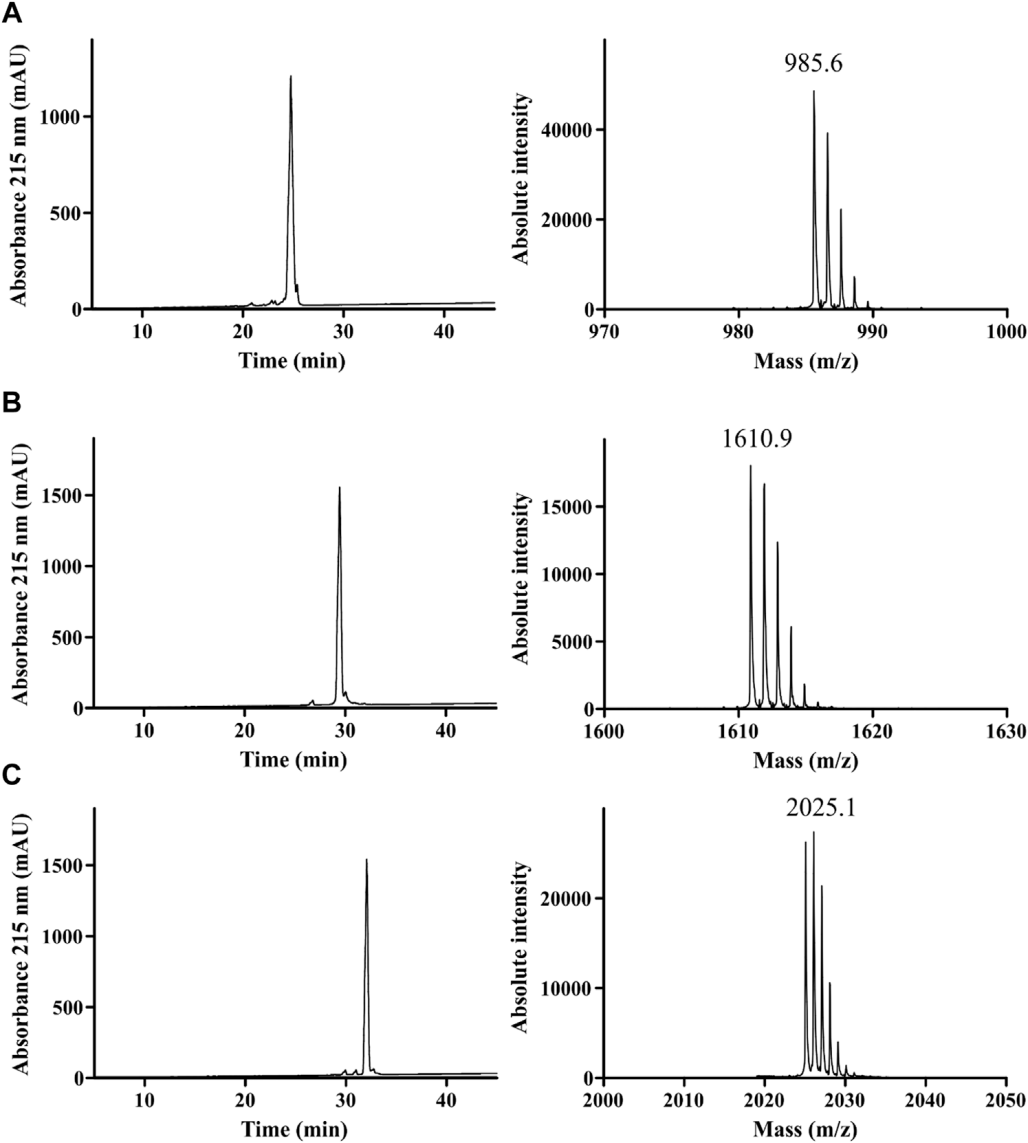
Quality control of synthesized blenniorphins. HPLC profiles (left) and MALDI mass spectra (right) of **(A)** blenniorphin 1–8, **(B)** blenniorphin 1–13 and **(C)** blenniorphin 1–17. Shown are monoisotopic [M + H]^+^ masses of peptides. The A215 chromatograms indicates a purity of >95%.

The *in silico* discovery approach identified blenny fish sequences which were highly similar to human dyn A 1–17. Accordingly, we hypothesized that blenny fish-derived peptides (Figure 1A) were ligands of a mammalian KOR. Previous structure-activity relationship studies on human dyn A 1–17 identified distinct dyn A fragments with significant activity at the KOR (Naqvi et al., 1998). Dyn A fragments 1–13 and 1-8 have been reported to bind to and activate the KOR with affinity and potency similar to dyn A 1–17. Thus, we opted to produce linear octa-, trideca- and heptadeca blenny fish-derived peptides via fluorenylmethoxycarbonyl (Fmoc)- and tert-butyloxycarbonyl (Boc)-based SPPS. These peptides, hereinafter termed as blenniorphins 1–8, 1–13, and 1–17 (Table 1), were manually assembled using standard side-chain protected amino acids and reagents described in previous published protocols (Cheneval et al., 2014). They were purified by semipreparative RP-HPLC and the final peptide products were characterized by mass spectrometry and analytical RP-HPLC. This confirmed their monoisotopic [M + H]^+^ of 985.6, 1610.9 and 2025.1 Da, respectively. The peptides had purities of 96, 98, and 97%, respectively, (Figure 2).

**TABLE 1:** Amino acid sequences of dynorphin A and blenniorphins.

Peptide	Sequence
dyn A 1–17	NH2-YGGFLRRIRPKLKWDNQ-COOH
blenniorphin 1–8	NH2-YGGFMRRV-COOH
blenniorphin 1–13	NH2-YGGFMRRVGRPEW-COOH
blenniorphin 1–17	NH2-YGGFMRRVGRPEWLVDS-COOH

### Blenniorphins Bind to and Activate the Mouse KOR

**FIGURE 3:**
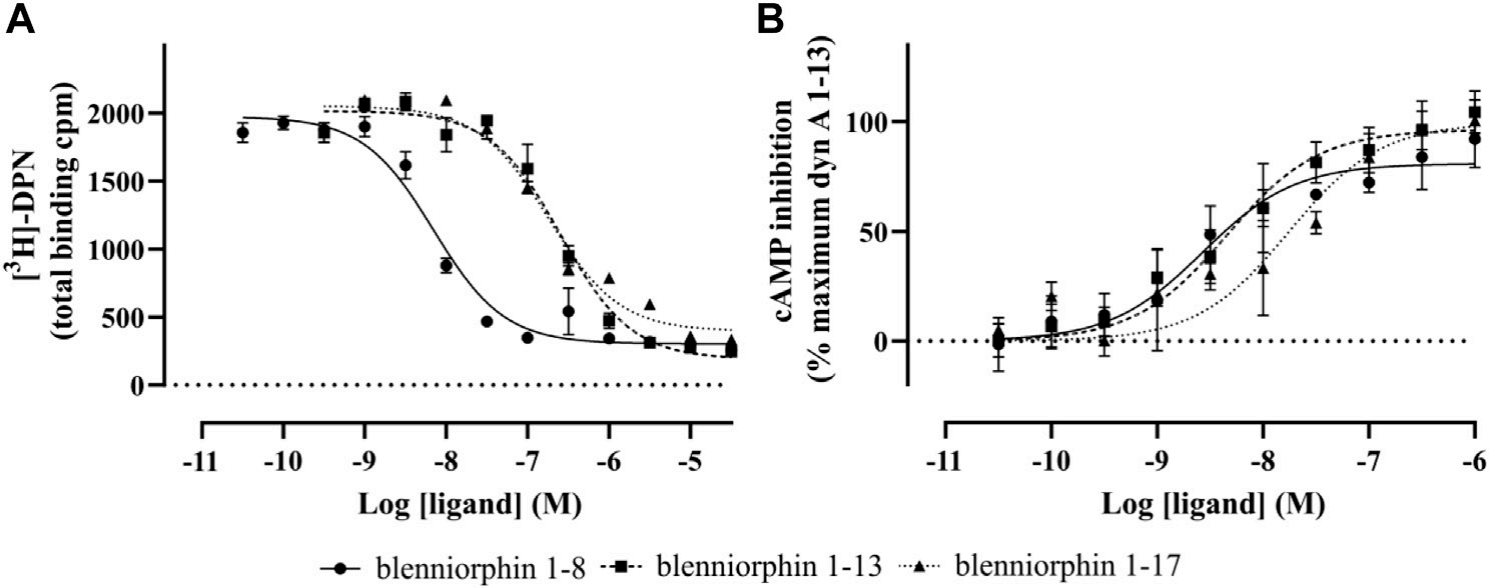
Radioligand displacement and functional cAMP assays of blenniorphins at the KOR. **(A)** Concentration-dependent displacement of [^3^H]-diprenorphine (DPN, 1 nM) was measured by blenniorphins 1–8, 1–13, and 1–17 using HEK293 cell membrane preparations stably expressing mouse KOR. Total binding is shown in counts per min (cpm) and data are presented as mean ± SD (*n* = 3). Nonspecific binding was determined from the curve fit and experimentally using 10 µM naloxone. **(B)** Functional cAMP assay was performed on HEK293 cells stably expressing mouse KOR. Forskolin (10 µM) was used for the activation of adenylyl cyclase. Data are normalized to 100% of dynorphin (dyn) A 1–13 measured at its saturating concentration (10 µM) and are shown as mean ± SD (*n* = 3).

Following chemical synthesis of blenniorphins, we next explored their ability to bind to and activate KOR by radioligand binding studies and by measuring their effect on cAMP inhibition, respectively. Radioligand binding studies revealed concentration-dependent displacement of [^3^H]-diprenorphine from murine KOR stably expressed in HEK293 cells by blenniorphins. Blenniorphin 1–13 and 1–17 bound to the KOR with *K*_i_ values of 116 ± 27 and 85.8 ± 15 nM, respectively. In contrast, the displacement curve for blenniorphin 1-8 was shifted to the left by more than one order of magnitude (*K*_i_ 3.30 ± 0.20 nM), thus demonstrating the highest affinity at the KOR (Figure 3A; Table 2). KOR couples to inhibitory G proteins leading to the inhibition of adenylyl cyclase and thus decrease in cellular cAMP levels. Hence, we determined the activity of blenniorphins by measuring cAMP inhibition (Figure 3B). The experiments showed that all fish-derived peptides stimulated the KOR as full/partial agonists with nanomolar potency. Blenniorphin 1-8 activated KOR with EC_50_ and E_max_ values of 3.60 ± 2.7 nM and 82.4 ± 9.8%, respectively, while blenniorphins 1–13 and 1–17 exhibited EC_50_ and E_max_ values of 5.00 ± 2.9 and 15.2 ± 9.4 nM and 98.5 ± 9.1% and 97.5 ± 11%, respectively, (Figure 3B; Table 2).

**TABLE 2:** Pharmacological data of blenniorphins at the KOR.

Ligand	Affinity	Potency/efficacy cAMP	
	K_i_ ± SD (nM)^c^	EC_50_ ± SD (nM)^c^	E_max_ ± SD (%)
dyn A 1–13^a^	0.0310 ± 0.0030	2.80 ± 0.70	100.0 ± 0.0
blenniorphin 1–8^b^	3.30 ± 0.20	3.60 ± 2.7	82.4 ± 9.8
blenniorphin 1–13^b^	116 ± 27	5.00 ± 2.9	98.5 ± 9.1
blenniorphin 1–17^b^	85.8 ± 15	15.2 ± 9.4	97.5 ± 11

a Binding and cAMP data for dynorphin (dyn) A 1–13 have been taken from previously published literature (Muratspahić et al., 2021).

b Data are from three independent experiments.

c Based on the experimental binding protocol (incubation of peptides for up to 1.5 h at 37°C), the addition of a protease inhibitor may further increase the affinity and potency, due to potential instability of the linear peptides.

### Blenniorphins Recruit β-Arrestin-2 at the KOR

**FIGURE 4:**
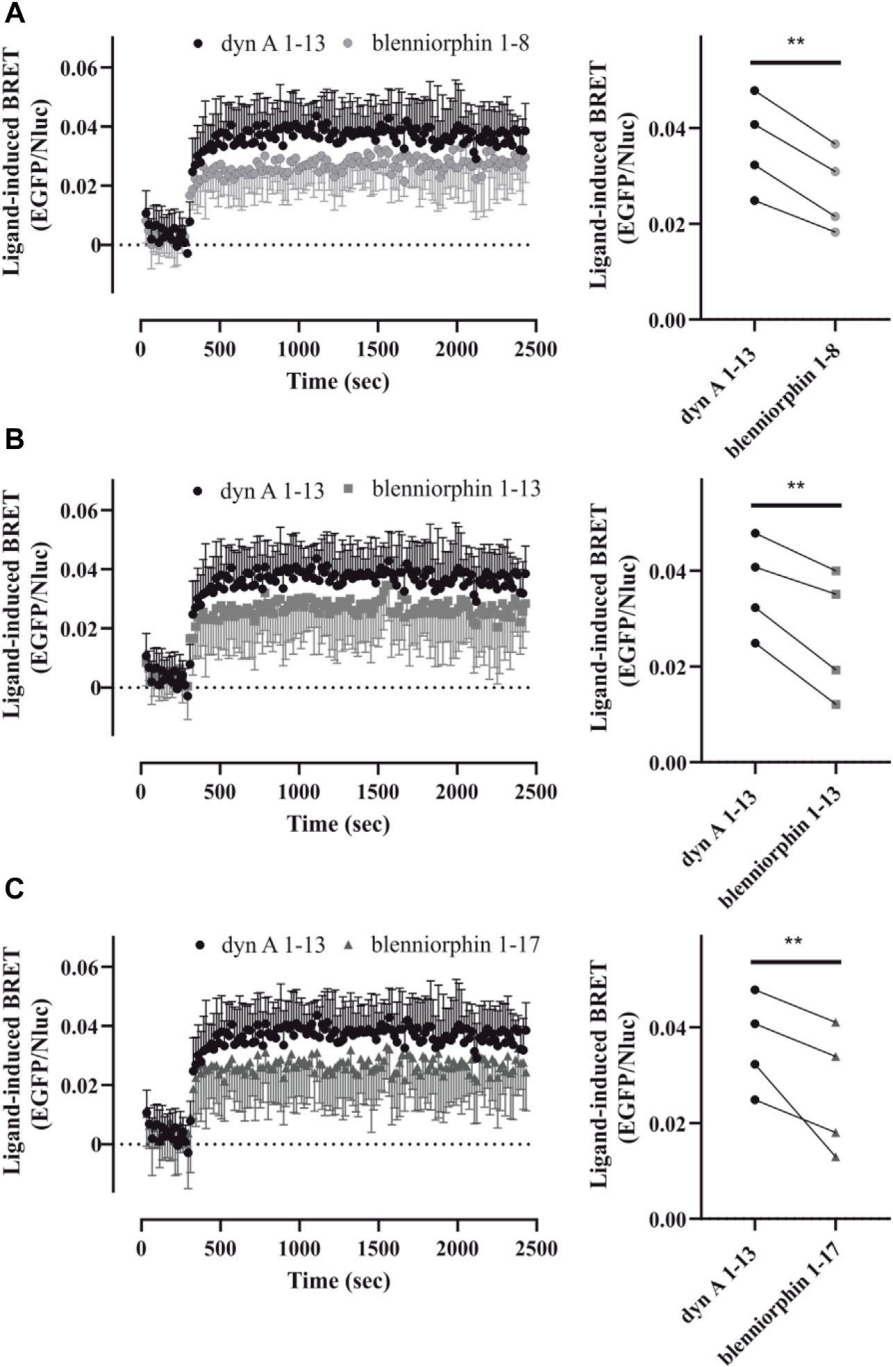
β-arrestin-2 recruitment by the KOR after stimulation by blenniorphins. **(A–C)** Recruitment of β-arrestin-2-Nluc by the EGFP-tagged KOR was measured over time by bioluminescence resonance energy transfer (BRET) prior to and after activation of the receptor by 10 µM blenniorphins 1–8 **(A)**, 1–13 **(B)** and 1–17 **(C)** (graphs on the left). In each instance, the HEK293 cells transiently co-expressing mouse KOR-EGFP and β-arrestin-2-Nluc were also stimulated with 10 µM dynorphin (dyn) A 1–13 that was used as the internal control. Data are shown as mean ± SD (*n* = 4). For each BRET dataset, ligand-induced arrestin recruitment by blenniorphins was quantified relative to the effect elicited by 10 µM dynorphin (dyn) A 1–13 and are shown as spaghetti plots (graphs on the right). Dyn A 1–13 controls are the same for **(A–C)**. Statistical significance was calculated by repeated measures ANOVA, followed by a Dunnet post-hoc comparison (**p < 0.01). EGFP, enhanced green fluorescence protein; Nluc, nano-luciferase.

β-arrestins are the cytosolic adapter proteins, which support the desensitization and internalization of many GPCRs including KOR (Appleyard et al., 1999; Li et al., 1999). Their recruitment to the receptor also initiates the activation of additional signaling pathways, which have been recently linked to the development of side effects associated with the agonist-induced activation of KOR (Brust et al., 2016a; Liu et al., 2019). We explored the ability of blenniorphins to recruit β-arrestins to KOR by conducting a BRET kinetics assay using HEK293 cells transiently co-expressing mouse KOR N-terminally fused to EGFP, and β-arrestin-2 N-terminally tagged with Nano-luciferase (Nluc). We used the endogenous human dyn A 1–13 as positive control given that it induces strong β-arrestin-2 recruitment at the KOR (Muratspahic et al., 2018). Intriguingly, blenniorphins recruited β-arrestin-2 with a time course comparable to that of human dyn A 1–13 (Figure 4). However, the extent of recruitment was significantly lower (by about 30%, *p < 0.01*) than that elicited by human dyn A 1–13 (100%) when measuring a saturating concentration of 10 µM (Figure 4).

## Discussion

Peptides are an established class of GPCR ligands (Davenport et al., 2020). To date, over 50 peptide GPCR drugs have been approved, notably for treating metabolic illnesses and cancer (Davenport et al., 2020; Muttenthaler et al., 2021). The majority of existing peptide therapeutics have been developed based on the structure-activity relationship studies of endogenous peptides (Davenport et al., 2020). Nature-derived peptides represent a growing niche for GPCR ligand discovery with over 100 nature-derived peptides hitherto identified as peptide GPCR ligands (Muratspahić et al., 2019). For instance, exendin-4 isolated from the Gila monster (*Heloderma suspectum*) is an example of a nature-derived peptide approved to treat diabetes mellitus type 2 by targeting glucagon-like peptide 1 receptor (Andersen et al., 2018).

Until recently, the isolation and extraction of such peptides was the only reliable approach for the identification of novel peptide GPCR ligands (Muratspahić et al., 2019). This strategy involved extensive fractionation and purification of peptides followed by their structural characerization (Muratspahić et al., 2019). Subsequently, these isolated peptides were screened in radioligand binding and functional studies (e.g., second messenger quantification and/or β-arrestin recruitment) using cells or tissues that either endogenously or exogenously expressed the GPCRs of interest (Kumari et al., 2015; Muratspahić et al., 2019). The pharmacological parameters such as affinity, potency and efficacy facilitated the identification of lead compounds which were then subject to structure-activity relationship studies to further optimize their pharmacological properties.

In the current genomics era with an ever-growing number of available sequences, genome mining has become an emerging strategy for the discovery of novel peptide GPCR ligands. In the present study, we used publicly available databases provided by the NCBI to identify novel fish-derived peptide ligands of the KOR by using human preprodynorphin precursor as a query. This analysis resulted in the discovery of over 300 dynorphin-like peptide sequences in distinct species of taxa of the bony fish. In fact, Casewell and others have recently constructed the transcriptomes from the venom gland of the blenny fish *M. atrodorsalis* and reported the pharmacological analysis of the venom extract (Casewell et al., 2017). Intriguingly, whereas the venom extract displayed significant activity in the functional cAMP assay in HEK293 cells expressing the DOR, it was inactive at the KOR and MOR (Casewell et al., 2017). On the other hand, pharmacological evaluation of purified dynorphin-like peptides, which we identified in our *in silico* analysis and which refer to as blenniorphins, revealed that they bind to and activate KOR in the low nanomolar range. In the binding assay blenniorphin 1-8 exhibited approximately 25- and 40-fold higher affinity for the KOR compared to blenniorphin 1–13 and blenniorphin 1–17, respectively. Previous findings on human dyn A 1–13 showed that lysine residues at position 11 and 13 are indispensable for its affinity and selectivity towards KOR (Snyder et al., 1992). Thus, the absence of lysine residues in blenniorphin-13 and blenniorphin-17 may account for their decreased affinity at the KOR. On the other hand, all three blenny fish-derived peptides demonstrated high potency and efficacy in the cAMP assay. Previous studies identified the arginine residues at the position 6 and 7 of human dyn A 1–13 to be crucial for its high potency in activating the KOR (Turcotte et al., 1984). All blenniorphins comprise these residues in their sequences. Hence, it is thus not surprising that they stimulate a mammalian KOR with a potency comparable to human dyn A 1–13 (Muratspahić et al., 2021). However, the rank order potency for the blenniorphans in binding vs. functional assays does not match. For instance, blenniorphins 1–8 and 1–13 exhibited higher potency values in the adenylyl cyclase assay as compared to their affinity values in the radioligand binding assay. This may be attributed to receptor reserve, which can be observed in certain functional GPCR assay types (Kelly, 2013).

Peptides have spurred interest in developing safer and more effective pain treatments notably because the prescription of opioids for pain relief and increasing availability of synthetic small molecule opioids including morphine and fentanyl have ignited the ongoing opioid crisis (Darcq and Kieffer, 2018). The KOR represents an excellent therapeutic target for developing analgesics with mitigated adverse effects, because its activation does neither contribute to the development of MOR-based respiratory depression nor addiction (Darcq and Kieffer, 2018). Over the past years, several strategies have been employed to discover and develop safer therapeutics targeting opioid receptors. One of these strategies relies on the design of molecules with reduced ability to cross the blood-brain barrier and targeting opioid receptors in the periphery. We have recently discovered that the sunflower trypsin inhibitor-1 from an extract of sunflower seeds acts as a ligand of the KOR (Muratspahić et al., 2021). Utilizing medicinal chemistry approaches, we improved the pharmacological properties of sunflower trypsin inhibitor-1 by designing helianorphin-19, a G protein-biased KOR agonist with peripheral analgesic activity and reduced centrally-mediated side effects (Muratspahić et al., 2021). The efforts to develop safer analgesics by targeting peripherally expressed KOR have recently culminated in the development of a peripherally restricted KOR tetrapeptide agonist, called difelikefalin, a and its clinical approval for the treatment of abdominal pain after surgery (Davenport et al., 2020). Hence, discovery of peptides targeting KOR is of special interest for combatting the opioid crisis and developing side effect-free pain therapeutics.

In this regard, biased ligands selectively activate pathways with beneficial clinical effects while decreasing signaling via pathways responsible for on-target side effects. This has sparked interest in the opioid receptor field to develop G protein-biased ligands as early studies identified β-arrestin signaling pathways to be instrumental for inducing deleterious side effects (Darcq and Kieffer, 2018). Using BRET as a further functional readout, we demonstrated that blenniorphins recruited β-arrestin-2 at a concentration of 10 μM, but their efficacy was lower than that of human dyn A 1–13. Despite the promising therapeutic potential of biased agonism, there is still debate about the contributions of β-arrestins in mediating opioid receptor-related side effects (Kliewer et al., 2019; Kliewer et al., 2020). In fact, Gillis and others have recently proposed that partial agonism rather than biased agonism per se may be attributable to the improvement of side effect profiles of opioids (Gillis et al., 2020a; Gillis et al., 2020b).

In conclusion, using genome mining we discovered novel fish-derived dynorphin-like peptides with intriguing pharmacological properties at the KOR. These peptides exhibited nanomolar affinity and potency for the KOR and altered β-arrestin-2 recruitment, as compared to the human endogenous dynorphin ligand. Our study reinforces potential of genome mining and fishes as a rich source for discovery of novel peptide ligands targeting GPCRs. These peptides may, in the future, serve as templates for the design of peptide-based analgesics with improved side effect profiles.

## Data Availability

The datasets presented in this study can be found in online repositories. The names of the repository/repositories and accession number(s) can be found in the article/Supplementary Material.
